# Identification of preoperative radiological risk factors for reoperation following percutaneous endoscopic lumbar decompression to treat degenerative lumbar spinal stenosis

**DOI:** 10.3389/fsurg.2022.1054760

**Published:** 2023-01-06

**Authors:** Aobo Wang, Tianyi Wang, Lei Zang, Ning Fan, Shuo Yuan, Fangda Si, Peng Du

**Affiliations:** Department of Orthopedics, Beijing Chaoyang Hospital, Capital Medical University, Beijing, China

**Keywords:** spinal stenosis, clinical outcome, minimally invasive surgery, endoscopy, paraspinal muscle, nomogram

## Abstract

**Background:**

This study aimed to identify radiological risk factors associated with reoperation after percutaneous transforaminal endoscopic decompression (PTED) for degenerative lumbar spinal stenosis (DLSS).

**Methods:**

The preoperative clinical data of 527 consecutive patients with DLSS who underwent PTED were retrospectively reviewed. Overall, 44 patients who underwent reoperation were matched for age, sex, body mass index, and surgical segment to 132 control patients with excellent or good clinical outcomes. Radiological characteristics were compared between the groups using independent sample *t*-tests and Pearson's chi-square tests. A predictive model was established based on multivariate logistic regression analysis.

**Results:**

The analyses revealed significant differences in the presence of lumbosacral transitional vertebra (LSTV, 43.2% vs. 17.4%, *p* = 0.001), the number of levels with senior-grade disc degeneration (2.57 vs. 1.96, *p* = 0.018) and facet degeneration (1.91 vs. 1.25 *p* = 0.002), and the skeletal muscle index (SMI, 849.7 mm^2^/m^2^ vs. 1008.7 mm^2^/m^2^, *p* < 0.001) between patients in the reoperation and control groups. The results of the logistic analysis demonstrated that LSTV (odds ratio [OR] = 2.734, 95% confidence interval [CI]:1.222–6.117, *p* < 0.014), number of levels with senior-grade facet degeneration (OR = 1.622, 95% CI:1.137–2.315, *p* = 0.008), and SMI (OR = 0.997, 95% CI:0.995–0.999, *p* = 0.001) were associated with reoperation after PTED. The application of the nomogram based on these three factors showed good discrimination (area under the receiver operating characteristic curve 0.754, 95% CI 0.670–0.837) and good calibration.

**Conclusion:**

LSTV, more levels with senior-grade facet degeneration, and severe paraspinal muscle atrophy are independent risk factors for reoperation after PTED. These factors can thus be used to predict reoperation risk and to help tailor treatment plans for patients with DLSS.

## Introduction

Degenerative lumbar spinal stenosis (DLSS) is one of the most diagnosed and treated pathologies of the spine (1). In the last two decades, as the use of minimally invasive techniques has become widespread, percutaneous transforaminal endoscopic decompression (PTED) has become a routine procedure for treating foraminal and lateral recess stenosis, and even central canal stenosis. In addition to herniated discs, PTED can be used to remove the hyperplastic ligament flavum and facet processes. This technique has been proven to be safe, clinically feasible, and effective (2).

However, unsatisfactory clinical outcomes are common in DLSS patients. Previous studies have shown that 3.5%–17.7% of patients with DLSS required reoperation after minimally invasive decompression (1, 3–6). Reoperation is commonly defined as an additional lumbar operation in a patient who has experienced a pain-free interval of at least one month after the initial PTED. After excluding complications related to the surgical technique, the main reasons for reoperation were restenosis or adjacent segment stenosis due to the progression of lumbar degeneration (4). Risk factors included age, obesity, decompression level, and spondylolisthesis (7, 8). However, we speculated that, in addition to these factors, some radiological parameters may also be helpful in predicting postoperative degeneration and reoperation.

Radiological evaluation included intervertebral disc, ligament flavum, facet joint, paraspinal muscle, and range of motion. Disc degeneration is considered the initial factor of segmental degeneration (9), while hypertrophy of the ligamentum flavum and facet joint is an important cause of nerve root compression. In recent years, the paraspinal muscles are gaining increasing attention. A decrease in paraspinal muscle function can lead to vertebral instability (10). Thus, degeneration of the above-mentioned structures has been considered significantly associated with poor surgical outcomes and revisions after traditional spinal fusion (11, 12). However, to the best of our knowledge, few studies have focused on the radiological characteristics of patients who underwent reoperation after PTED for DLSS.

In the present study, we performed a retrospective, matched case-control study to investigate the association between radiological parameters and reoperation after PTED and to build a model to predict reoperation risk based on the verified risk factors.

## Methods

### Patient population

We retrospectively reviewed the clinical and imaging data of patients with lateral recess or foraminal stenosis who underwent single-level PTED at our institution between January 2016 and July 2020. The inclusion criteria were as follows: (1) age >40 years, (2) unilateral symptoms of lateral recess or foraminal stenosis, radiological findings consistent with clinical symptoms in terms of pain location. (3) failure of conservative treatment for at least three months. The corresponding exclusion criteria were as follows: (1) spondylolisthesis greater than grade-1, (2) multilevel symptomatic lumbar stenosis; (3) symptoms caused only by disc herniation; (4) significant residual pain or other short-term complications after PTED; (5) a follow-up time of less than 24 months or loss to follow-up; (6) a history of lumbar surgery; (7) nondegenerative lumbar diseases such as tumor, infection, and trauma; and (8) insufficient clinical or imaging data.

Overall, we identified 527 eligible patients, of whom 44 underwent additional PTED or spinal fusion at the same or adjacent level. Information on patients who underwent reoperation is summarized in Table 1. A control group of patients with excellent or good clinical outcomes were propensity score-matched to the reoperation group in terms of age, sex, body mass index (BMI), and surgical segment in a 1:3 manner. The demographic data of the patients in the two groups are summarized in Table 2. This study was performed in accordance with the Declaration of Helsinki, and approval was obtained from the institutional ethics committee.

**Table 1:** Information of patients who underwent reoperation.

	Number of patients
**Indication of reoperation (compared to the initial operation)**	
Surgical level restenosis	33 (75.0%)
Adjacent level stenosis	11 (20.0%)
**Stenosis location before reoperation**	
Central canal stenosis	11 (25.0%)
Foraminal stenosis	15 (34.1%)
Lateral recess stenosis	18 (40.9%)
**Surgical procedure**	
Percutaneous transforaminal endoscopic decompression	31 (70.5%)
Posterior lumbar interbody fusion	11 (25.0%)
Endoscopic transforaminal lumbar interbody fusion	2 (4.5%)

**Table 2:** Demographic and clinical characteristics of the included patients.

	Reoperation group (*n* = 44)	Control group (*n* = 132)	*p*
Age (years)	65.4 ± 12.2	64.6 ± 11.0	0.685
Gender (male/female)	22/22	70/62	0.862
Duration of symptoms (months)	59.0 ± 80.6	57.0 ± 75.1	0.879
Body mass index (kg/m2)	25.9 ± 3.1	25.7 ± 3.8	0.837
Surgical level (number of patients)			1.000
L3/4	2	8	
L4/5	33	99	
L5/S1	9	25	
Duration of operation (minutes)	88.7 ± 12.7	90.4 ± 13.9	0.562
Duration of follow-up (months)	44.5 ± 14.5	45.4 ± 10.0	0.722

### Surgical methods

All PTED procedures were performed by a senior surgeon with experience of more than 100 percutaneous endoscopic procedures. In patients with multilevel radiographic stenosis, nerve root blocking was performed to determine the level of responsibility. PTED was further performed as follows: The entire procedure was performed with the patient in the prone position, and under local anesthesia. The entry point was set at 10–14 cm lateral to the spinal midline at the index intervertebral level. A puncture needle was inserted into the superior articular process (SAP). An 8 mm working cannula was placed in contact with the surface of the SAP after expending the surgical approach using serial hollow cannulas. A trephine was then used to remove the capsule and the ventral side of the SAP. Decompression was performed using continuous irrigation under direct vision. The osteophyte, thickened ligament flavum, perineural fat, degenerated annulus fibrosus, and nucleus pulposus were removed to ensure complete decompression.

### Data collection and assessment

The demographic and clinical data, including age, sex, body mass index (BMI), smoking status, duration of symptoms, surgical level, duration of surgery, and follow-up duration, were recorded for all enrolled patients. Radiological data included disc degeneration grade, spinal stenosis grade, facet joint degeneration grade, lumbar lordosis, disc height index (DHI), disc wedging angle, facet orientation, facet tropism, paraspinal muscle degeneration, sagittal range of motion (sROM), Modic changes, and lumbosacral transitional vertebra (LSTV).

**Figure 1:**
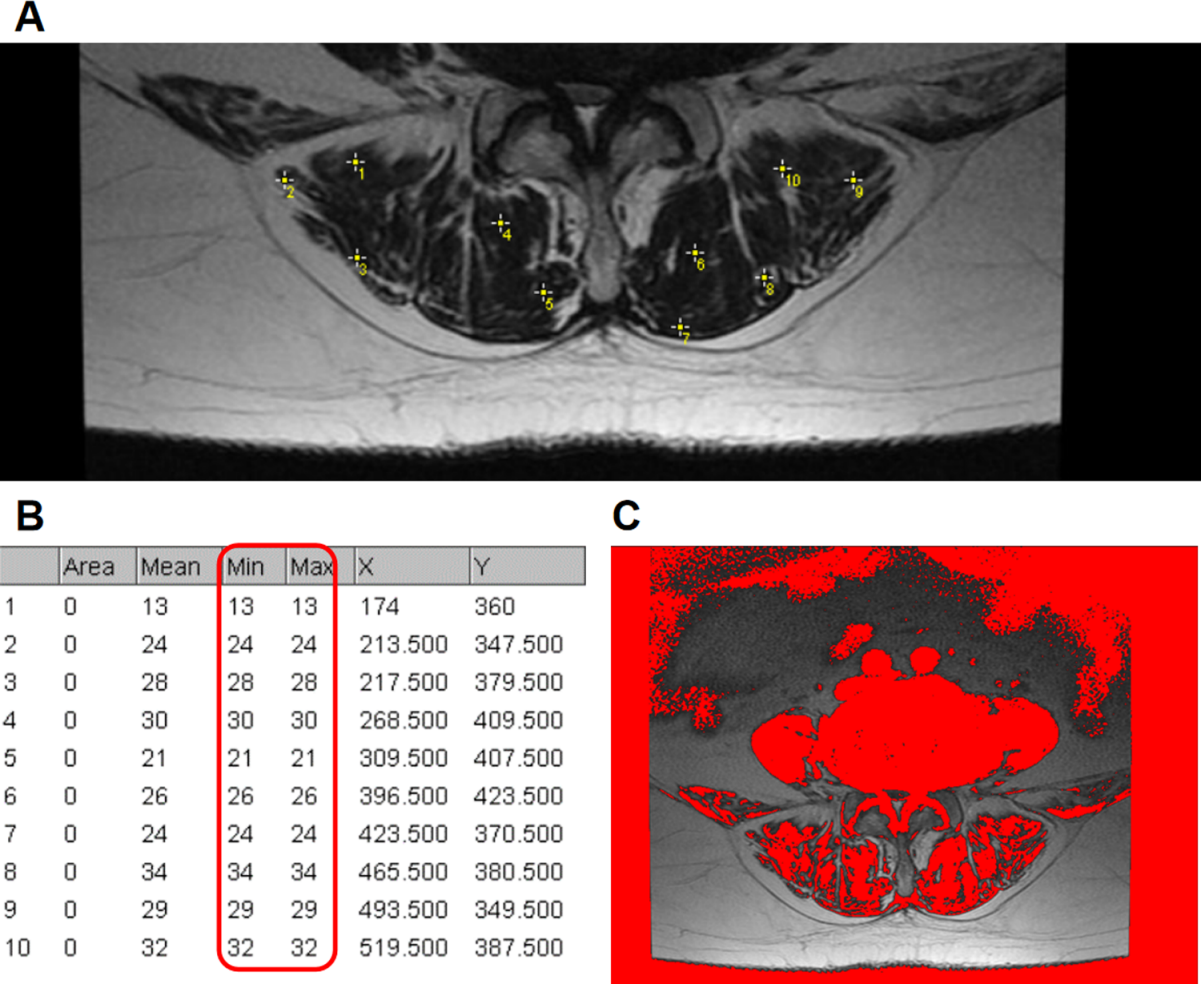
Use of the “Multi-point” tool to determine the threshold to distinguish lean muscle tissue from fat tissue. (**A**) Ten sample points without any visible pixel of fat tissue within the bilateral paraspinal muscle were selected. (**B**) The maximum signal intensity of the ten points was determined as the threshold. (**C**) An example axial T2 weighted MR image of the functional cross-sectional area of the muscles (red area), using the threshold method.

**Figure 2:**
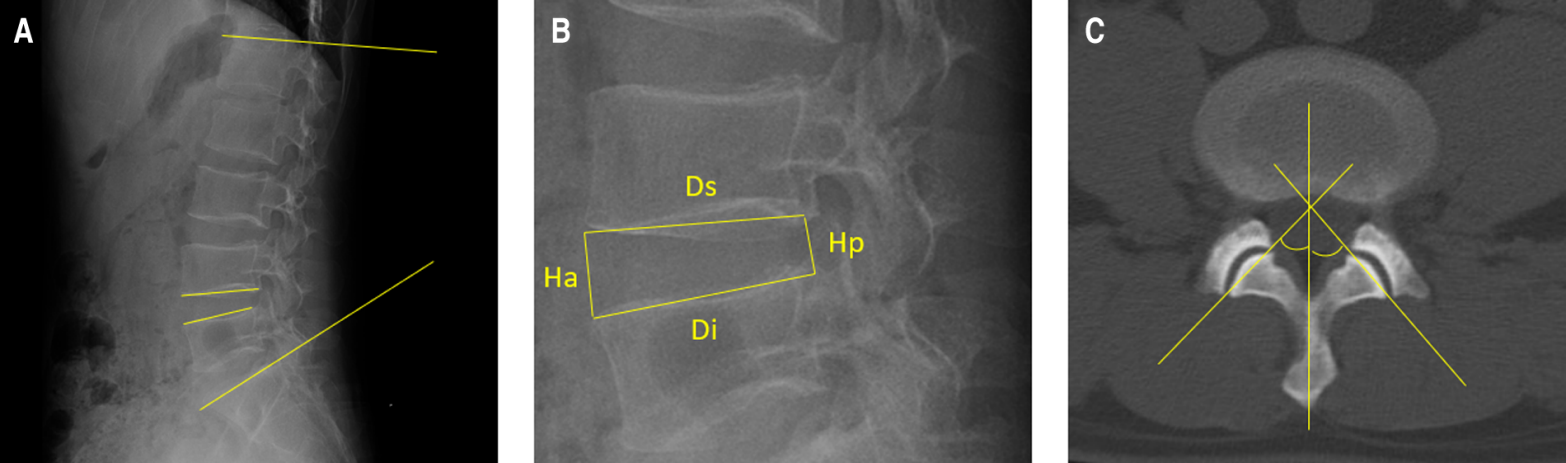
Illustration of the measurements of the radiological parameters. (**A**) Lumbar lordosis is defined as the angle between the superior endplates of L1 and S1. The disc wedging angle is defined as the angle between the lower endplate of the upper vertebra and the upper endplate of the lower vertebra. Additionally, the sagittal range of motion is defined as the absolute difference between the disc wedging angles in flexion and extension positions. (**B**) Disc height index is calculated with the equation: [(anterior disc height + posterior disc height)/(superior disc depth + inferior disc depth)]×100. (**C**) A line is drawn to connect the anterior and posterior margins of the superior articular process. The other line represents the midsagittal line. The facet orientation is calculated as the mean of the facet angle between the right and left sides. The facet tropism is the absolute difference between the two sides.

The disc degeneration grade was evaluated using sagittal T2-weighted magnetic resonance imaging (MRI) according to Pfirrmann (13). Spinal stenosis grade was evaluated using axial T2-weighted MRI according to Schizas (14). Facet joint degeneration was evaluated using axial computerized tomography images according to Weishaupt (15). The grade of surgical-level degeneration of the aforementioned structures and the number of lumbar levels with senior-grade degeneration were recorded. The skeletal muscle index (SMI) (16) was used to assess paraspinal muscle degeneration as body height has been proven to be related to paraspinal muscle mass. SMI was calculated as the bilateral functional cross-sectional area (Figure 1) of the paraspinal muscle at the mid-disk of the L4/5 level (expressed as millimeters squared)/the square of the patient's height (expressed as meters squared). Endplate degeneration was evaluated according to Modic changes. The measurements of disc wedging angle, DHI, sROM, lumbar lordosis, facet orientation, and facet tropism are shown in Figure 2.

### Statistical analysis

For statistical analyses, data were analyzed using statistical software (SPSS version 20.0, for Windows, IBM) and R 4.1.3 (The R Foundation for Statistical Computing, Vienna, Austria). All demographic, clinical, and radiological data were compared between the two groups using independent sample *t*-tests and Pearson's chi-square tests. Variables with *p*-values less than 0.10 were further included in the multivariable logistic regression analysis. Variance inflation factor (VIF) was used to diagnose collinearity. Based on the results of the regression analysis, a nomogram for reoperation probability was constructed, and its performance was assessed using the area under the receiver operating characteristic curve and a visual calibration plot. All continuous values are presented as the mean ± standard deviation. Statistical significance was set at *p* < 0.05.

## Results

A total of 176 patients were included in this study which aimed to identify radiological predictors for reoperation after PTED (44 in the reoperation group and 132 in the control group). Demographic and clinical characteristics of the enrolled patients are shown in Table 2. There were no significant differences in age, sex, duration of symptoms, BMI, surgical level, duration of operation, or duration of follow-up between the two matched groups. Among the patients in the reoperation group, the mean reoperation time was 16.8 months.

The results of the univariate analyses of radiological parameters are shown in Table 3. Compared with patients in the control group, patients in the reoperation group had a higher risk of LSTV (43.2% vs. 17.4%, *p* = 0.001), greater number of levels with senior-grade disc degeneration (2.57 vs. 1.96, *p* = 0.018) and facet degeneration (1.91 vs. 1.25, *p* = 0.002), and a smaller SMI (849.7 mm^2^/m^2^ vs. 1008.7 mm^2^/m^2^, *p* < 0.001). There were no significant differences in other radiological characteristics between the two groups (*p* > 0.05).

**Table 3:** Univariate analyses of the radiological parameters between patients in the reoperation and control groups.

	Reoperation group (*n* = 44)	Control group (*n* = 132)	*p*
Lumbar lordosis (°)	32.6 ± 11.4	30.9 ± 10.6	0.371
Modic changes (*n*)			0.630
No changes	28	92	
Type 1	6	12	
Type 2	10	28	
LSTV (yes/no)	19/25	23/109	0.001*
Sacralization	15	20	
Lumbarization	4	3	
Grade of surgical-level spinal stenosis			0.585
Low grade (A1–A4)	30	84	
Senior grade (B–D)	14	48	
Number of levels with senior grade stenosis	0.591 ± 0.923	0.705 ± 0.779	0.425
Grade of surgical-level disc degeneration			0.424
Low grade (I–III)	8	24	
Senior grade (IV, V)	36	108	
Number of levels with senior grade disc degeneration	2.57 ± 1.53	1.96 ± 1.18	0.018*
Disc wedging angle (°)	7.68 ± 3.44	7.40 ± 3.12	0.616
DHI	24.4 ± 5.3	25.5 ± 4.0	0.128
Grade of surgical-level facet degeneration			0.089
Low grade (0, I)	12	55	
Senior grade (II, III)	32	77	
Number of levels with senior grade facet degeneration	1.91 ± 1.27	1.25 ± 0.93	0.002*
Facet orientation (°)	34.7 ± 10.3	35.4 ± 8.1	0.658
Facet tropism (°)	7.05 ± 5.35	6.48 ± 3.58	0.514
SAPA (mm^2^)	130.5 ± 17.1	127.9 ± 12.5	0.270
SMI (mm^2^/m^2^)	849.7 ± 181.0	1008.7 ± 251.2	<0.001*
sROM (°)	7.91 ± 3.92	6.39 ± 2.92	0.268

LSTV, lumbosacral transitional vertebrae; DHI, disc height index; SAPA, superior articular process cross-sectional area; SMI, skeletal muscle index; sROM, sagittal range of motion.

* Statistically significant at *p* < 0.05.

The grade of surgical-level disc degeneration, LSTV, number of levels with senior-grade disc degeneration, number of levels with senior-grade facet degeneration, and SMI were included in further multivariate logistic regression analyses. The collinearity test revealed no collinearity among the variables (VIFs < 10). Multivariate logistic regression analysis demonstrated that LSTV (odds ratio [OR] = 2.734, 95% confidence interval [CI]:1.222–6.117, *p* < 0.014) and number of levels with senior-grade facet degeneration (OR = 1.622, 95% CI:1.137–2.315, *p* = 0.008) were independent risk factors for reoperation after PTED. SMI (OR = 0.997, 95% CI:0.995–0.999; *p* = 0.001) was a protective factor for reoperation after PTED (Table 4).

**Table 4:** Multivariate regression model of the predictors for reoperation following percutaneous transforaminal endoscopic decompression.

	Significance	Odd ratio	95% confidence interval
LSTV (yes)	0.014*	2.734	1.222–6.117
Number of levels with senior grade facet degeneration (every 1 level)	0.008*	1.622	1.137–2.315
SMI	0.001*	0.997	0.995–0.999
Number of levels with senior grade disc degeneration (every 1 level)	0.324		
Grade of surgical-level facet degeneration (every 1 grade)	0.605		

LSTV, lumbosacral transitional vertebrae; SMI, skeletal muscle index.

* Statistically significant at *p* < 0.05.

**Figure 3:**
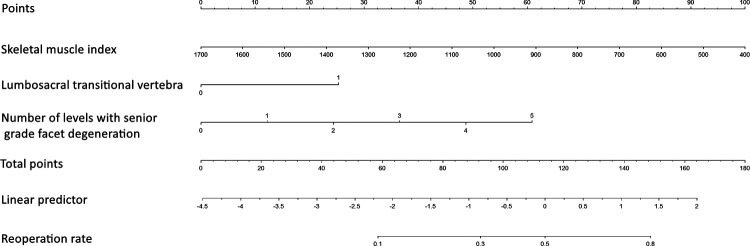
Nomogram for predicting reoperation risk in patients who underwent percutaneous transforaminal endoscopic lumbar decompression.

**Figure 4:**
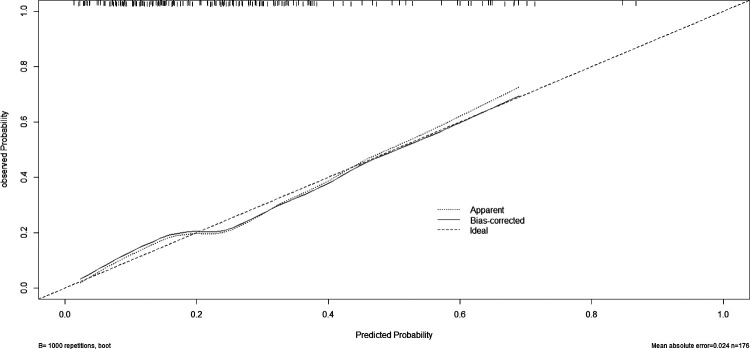
Calibration curve of the predictive model.

**Figure 5:**
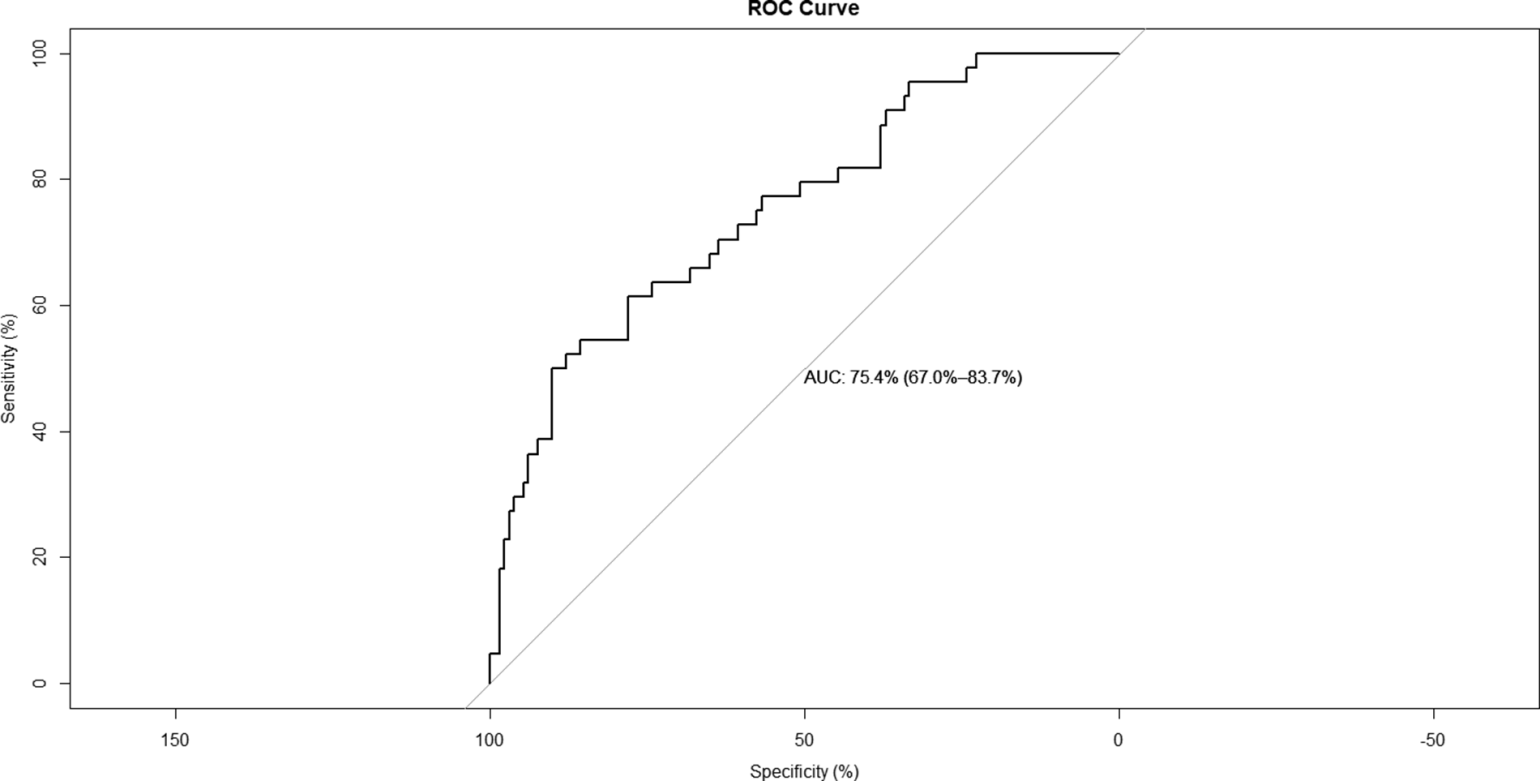
Receiver operating characteristic curve of the predictive model.

A nomogram for predicting reoperation after PTED was constructed based on radiological factors selected by logistic regression (Figure 3). The calibration curve of the nomogram indicated that the predicted probability agreed well with the actual recurrence (Figure 4). The area under the receiver operating characteristic curve of the model was 0.754 (95% CI,0.670–0.837), which shows the reliability of this model (Figure 5).

## Discussion

PTED is a widely used surgical technique for DLSS treatment. However, recurrent postoperative symptoms and reoperations are disturbing problems, and the risk factors for reoperation after PTED have not been fully investigated. In this retrospective case-control study, we compared the radiological characteristics between patients who underwent reoperation and those with satisfactory outcomes. Patients in the reoperation group achieved remission immediately after primary surgery. Thus, the indication for reoperation was the progression or recurrence of lumbar stenosis, rather than surgical complications. The results of multivariate logistic regression analyses showed that the presence of LSTV, the number of levels with senior-grade facet degeneration, and a smaller SMI were independent risk factors for reoperation after PTED. A nomogram based on these radiological parameters could predict the risks of reoperation occurrence after PTED, with optimal discrimination and excellent calibration.

Urakawa (17) previously investigated factors associated with reoperations following posterior lumbar decompression and found that age <70 years and symptomatic neurogenic claudication were significantly associated with secondary fusions. Cummins (18) further reported that female sex and history of lumbar decompression were risk factors for revision surgery. Yin (7) concluded that age and BMI could influence recurrence rate. Our previous study also confirmed that age was a risk factor for reoperation after PTED (19). However, we found that the associations between age, sex, and reoperation were ambiguous in the existing literature. The conflicting results among previous studies may reflect differences in cultural and socioeconomic situations. Therefore, in this study we focused on the radiological characteristics, which are relatively objective indicators, and the influence of demographic characteristics on reoperation was decreased by matching. However, previous studies have proposed that risk factors might differ among subgroups (20). The main objects of this study were geriatric patients, and further studies stratified by factors such as age and sex based on a larger sample size, are required in the future.

In this study, the number of levels with senior-grade facet degeneration and the presence of LSTV were found to be risk factors for reoperation after PTED. Facet degeneration is considered an adaptive change of increased compression (21). Articular processes tend to maintain lumbar stability through hyperplasia and lengthening of the articular surface (22). Therefore, multilevel facet degeneration reflects severe lumbar aging and abrasion. In addition, hypertrophy of the superior articular process itself is a significant cause of nerve root compression (9), increasing the risk of progression of foraminal and lateral recess stenosis.

LSTV is a common anatomical variant, with a prevalence of 7%–36% (23). Although the biomechanical influence of the LSTV has not been fully explained, many scholars believe that individuals with LSTV are at a higher risk of degenerative diseases in each lumbar level, especially at the adjacent cephalad level (23–25). One potential explanation for this is that LSTV leads to uneven loads on the lumbar spine, resulting in hypermobility and an increase in segmental stress (26). Additionally, the enlarged transverse process and sacral ala are more likely to impinge on nerve roots and cause relative symptoms (27). Furthermore, there are several subtypes of LSTV. Some researchers believe that sacralization can lead to a more abnormal increase in segmental stress, just like the pathological changes after a spinal fusion (24, 28). Besides, unilateral LSTV may lead to asymmetric stress on the lumbar spine and thus more severe degeneration compared with bilateral LSTV (28). However, the influence of these subtypes is still under controversial and most of the previous studies tended to regard LSTV as a unified feature (24, 25). In this study, the small sample size limits our further analysis of LSTV. Larger studies are needed to investigate the effect of various types of LSTV on the prognosis of lumbar spinal stenosis.

Additionally, we found that patients who underwent reoperation had higher levels of senior-grade disc degeneration, which may have a similar effect on the lumbar spine as facet degeneration and LSTV (29). However, this is not a risk factor for reoperation, which may be because disc degeneration is common in geriatric patients.

**Figure 6:**
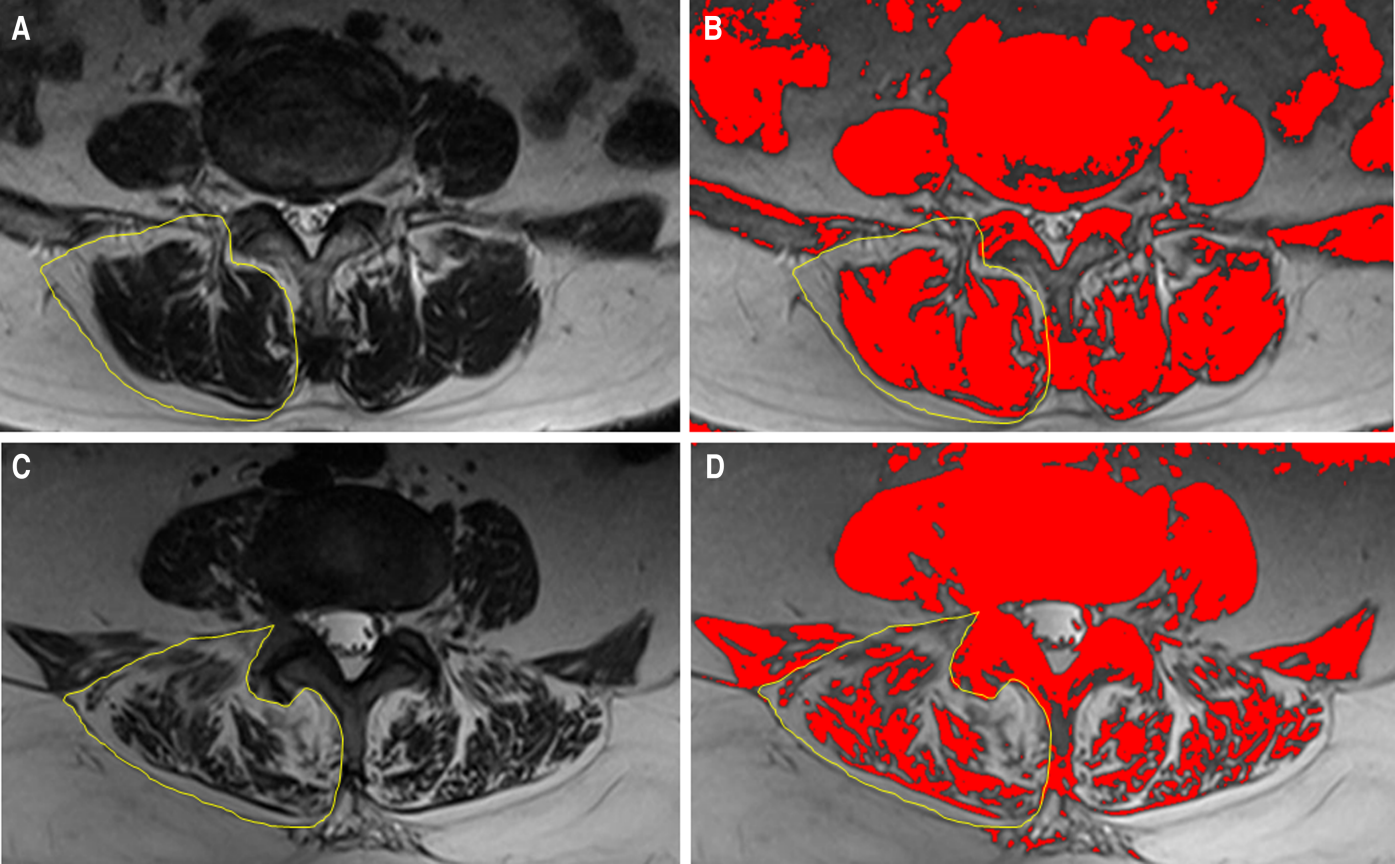
Illustration of the comparison between patients who underwent reoperation after PTED and controls. The circled area indicates the unilateral paraspinal muscle. (**A,B**)Preoperative paraspinal muscle mass of a 62-year-old woman in the control group. (**C,D**)Preoperative paraspinal muscle mass of a 65-year-old woman in the reoperation group.

Paraspinal muscle degeneration is a complex process which is significantly associated with spinal degeneration, deformity, and dysfunction, which has been a hot topic in the past decade. In many previous studies, the paraspinal muscle was evaluated as the sum of the multifidus muscle and erector spinae (30), since these act synergistically in most instances, and the boundaries between them are unclear in some cases. The paraspinal muscles attach directly to the lumbar vertebrae and play a significant role in the rotation, flexion, extension, and maintenance of lordosis of the lumbar spine (31, 32). In patients with paraspinal muscle atrophy, the role of muscle fibers as stabilizers is weakened, further causing increased spinal axial loading (10, 33). Additionally, the loss of muscle function may lead to a degree of sagittal and coronal misalignment. These changes can lead to disc degeneration, ligament flavum hypertrophy, and facet joint osteoarthritis, all of which cause lumbar stenosis (34, 35). Thus, paraspinal muscle atrophy may accelerate the progression of lumbar degeneration (36), which could explain the difference in paraspinal muscle mass between the groups (Figure 6).

In general, all the risk factors for reoperation identified in this study were associated with weakening of the support structure and an increase in axial loading. Although these changes are difficult to reverse, our results nevertheless point to several tactics that can be implemented in clinical practice. First, for high-risk patients, cautious selection of the appropriate treatment modality and informing of the possibility of relapse of the disease in advance are necessary. Second, it has been demonstrated that increased mechanical loading due to strenuous exercise and overstrain can lead to poor clinical outcome (37). The high-risk patients identified in this study were less capable of withstanding lumbar stress. Therefore, such individuals should pay special attention to avoiding overwork in their daily lives. Third, among the risk factors for reoperation in this study, paraspinal muscle degeneration was the only factor that could be ameliorated by intervention. Previous studies have shown that physical therapy such as trunk exercises and cupping can increase multifidus muscle thickness and improve surgical outcomes (38, 39).

Interestingly, no radiological characteristics at the surgical level, such as the grade of disc degeneration and presence of central canal stenosis, were found to be risk factors for reoperation after PTED in this study. This result differs from some previous findings (40). We speculate that this difference may be explained by the following points. First, with the development of a minimally invasive technique, PTED can achieve satisfactory results in the treatment of various lumbar degenerative diseases (2). After a detailed preoperative evaluation, a surgeon who has surpassed the learning curve can adequately decompress the spinal canal and nerve root in most cases. Second, spondylolisthesis is considered a risk factor for reoperation (41); however, it was excluded from the study. For geriatric DLSS patients without severe instability, lumbar discs and facet joints can achieve an instability-to-restabilization process with degeneration and reconstruction of the lumbar spine (42). Thus, segmental stability can be maintained even with severe degeneration, and segmental factors may have a limited effect on the progression of lumbar degeneration. Similarly, the level of Modic changes is not significantly associated with reoperation in this study. The presence of Modic changes has been considered to indicate local instability and therefore a risk factor for recurrent disc herniation postoperatively (20, 43). However, as is discussed above, this influence may not be as significant in geriatric DLSS patients. This conclusion is consistent with other previous findings (20, 44). Notably, due to the mechanical defect, patients with Modic changes, especially type 1, have a trend of deterioration in postoperative back pain, in which endoscopic procedures may be less effective (45).

This study had several limitations. First, the study was retrospective, based on a relatively small sample size, and the control group was established using propensity score matching. Second, the decision to reoperate was influenced by many factors, including osteoporosis, sociopsychological conditions, and personal choices. However, these confounders were not considered in the present study. Third, we identified several risk factors associated with reoperation after PTED; however, we could not determine the causal relationship between them. Therefore, long-term longitudinal studies are warranted. Fourth, although SMI has been recognized as the most accurate indicator of paraspinal muscle atrophy, it is a complicated continuous variable that results in a small odds ratio. As such, there is a need for a scientific grading system for paraspinal degeneration.

## Conclusion

This study confirmed that the presence of LSTV, more levels with senior-grade facet degeneration, and severe paraspinal muscle atrophy were independent risk factors for reoperation after PTED. A nomogram based on these factors could be applied in clinical practice to predict the need for reoperation and help improve individualized treatment planning.

## Data Availability

The datasets generated during and/or analyzed during the current study are available from the corresponding author on reasonable request.
